# Effectiveness of bronchial thermoplasty in patients with asthma exhibiting overweight/obesity and low quality of life

**DOI:** 10.1016/j.waojou.2023.100756

**Published:** 2023-03-20

**Authors:** Kenta Nishi, Chie Yoshimura, Kyohei Morita, Ryoichi Ishikawa, Erika Toyokura, Tadao Nagasaki, Hisako Matsumoto, Yasuo Nishizaka

**Affiliations:** a Department of Respiratory Medicine, Osaka Red Cross Hospital, Osaka, Japan; b Department of Respiratory Medicine, Kyoto University Graduate School of Medicine, Kyoto, Japan; c Department of Respiratory Medicine and Allergology, Kindai University Faculty of Medicine, Osaka, Japan

**Keywords:** Bronchial thermoplasty, Severe asthma, Obesity, Body mass index, Phenotype

## Abstract

Bronchial thermoplasty (BT) is effective in some severe asthma patients; however, the specific asthma phenotypes that produce a good response to BT are not fully understood. Clinical data were retrospectively reviewed in severe asthma patients who underwent BT at a single institution in Japan. At the follow-up assessment, the Asthma Quality of Life Questionnaire (AQLQ) scores (*P* = 0.003), maintenance oral corticosteroid doses (*P* = 0.027), and exacerbation frequency (*P* = 0.017) were significantly improved, while prebronchodilator-forced expiratory volume in 1 second (% predicted) did not significantly change (*P* = 0.19). When we grouped the patients into 2 groups according to their body mass index levels, the AQLQ scores were more improved in patients with overweight/obesity than those with normal weight (*P* = 0.01). This study showed that patients with non-controlled severe asthma exhibiting overweight/obesity and low quality of life had potential benefits from BT.

To the Editor

Bronchial thermoplasty (BT), which improves asthma-specific quality of life (QOL) and asthma exacerbation,^1^ is an additional non-pharmacological treatment for severe asthma. Previous reports in real-world setting including the elderly, smokers, and patients with airflow obstruction also showed clinical effectiveness of BT treatment.^2^^,^^3^ However, the specific asthma phenotypes that produce a good response to BT treatment remain unknown. Most previous studies, which reported the effectiveness of BT, included patients with an average body mass index (BMI) of approximately 30 kg/m^2^.^2^, ^3^, ^4^ To the best of our knowledge, no studies have examined the difference in effects of BT in a cohort stratified by BMI. According to the current guideline, BT treatment can be considered in patients with non-type 2 airway inflammation or those with a poor response to type 2-targeted biologics.^5^ The distinct phenotype of asthma with obesity is recognized among patients with non-type 2 asthma.^6^ In the clinical experience, we observed that BT was effective for patients with severe asthma who were overweight/obese. Therefore, we assessed the effect of BT treatment on various clinical indices in patients stratified by BMI.

In this retrospective and observational cohort analysis, we used data from patients with severe asthma who underwent BT at a single institution in Japan between September 2017 and October 2019. BT was indicated by pulmonologists for adult patients with severe persistent asthma who did not achieve good control despite optimized treatment and who agreed to receive BT treatment. We collected clinical data recorded at the last visit before BT (baseline assessment) and at 12 months after BT (follow-up assessment). Exacerbation was defined as worsening in asthma control, thereby requiring systemic corticosteroids or doubling or more the daily doses of baseline maintenance oral corticosteroid (mOCS) for at least 3 days. The Asthma Quality of Life Questionnaire (AQLQ) consists of 32 items scored on a seven-point Likert scale. In this study, the average of total scores was used. The minimal clinically important difference was a 0.5-point increase.^7^

All statistical data were analyzed using JMP version Pro 15 (SAS Institute Inc., Tokyo, Japan). Data are described as means ± SD or median (interquartile range). In comparing between groups, we used Student's *t*-test or Wilcoxon rank-sum test, or χ^2^ test, as appropriate. Correlations between 2 variables were examined using Pearson's correlation coefficient. Baseline and follow-up assessments were compared by paired *t*-test or Wilcoxon signed-rank test. Furthermore, changes in paired data between two groups were compared by Multivariate Analysis of Variance (MANOVA). We considered *P* < 0.05 as statistically significant.

BT was provided to 23 patients. However, 2 of them were excluded because their follow-up data were unavailable. Table 1 summarizes the baseline characteristics of eligible patients (*n* = 21). We found that 28.6% (*n* = 6) were >65 years, 57.1% (*n* = 12) had blood eosinophil counts <150/μL, and 38.1% (*n* = 8) had prebronchodilator-forced expiratory volume in 1 second (FEV_1_) (% predicted) < 60%. Additionally, all the patients received Global Initiative for Asthma Step 5 treatments.^5^ Six patients had received biologics treatment before BT (*n* = 3, omalizumab over 3 years; *n* = 1, mepolizumab for 5 months; *n* = 2, benralizumab less than 3 months). At the follow-up assessment of the overall population, the AQLQ scores (points) (pre: 4.3 ± 1.2, post: 5.4 ± 1.2; *P* = 0.003), mOCS doses (mg/day) [pre: 3.8 (2.5–5.0), post: 2.5 (1.0–4.5); *P* = 0.027], exacerbation frequency (/1 year) [pre: 1.0 (0.5–3.5), post: 0.0 (0.0–2.0); *P* = 0.017], and forced vital capacity (FVC) (% predicted) (pre: 94.0 ± 20.1, post: 98.4 ± 19.4; *P* = 0.039) (Supplementary Fig. 1a–d) significantly improved, while FEV_1_ (% predicted) did not significantly change (pre: 76.6 ± 24.8, post: 79.6 ± 26.4; *P* = 0.19) (Supplementary Fig. 1e). In most patients, the AQLQ scores increased by 0.5 points or more (*n* = 15, 71.4%), exacerbations were reduced (*n* = 13, 61.9%), and the mOCS doses decreased (*n* = 8, 72.7%). The baseline AQLQ scores were inversely associated to the delta AQLQ (Supplementary Fig. 2).

**Table 1:** Baseline characteristics of patients stratified by BMI.

	Overall (*n* = 21)	BMI <25 kg/m^2^ (*n* = 11)	BMI ≥25 kg/m^2^ (*n* = 10)	*P* value^†^
Age (years)	56.8 ± 12.3	58.3 ± 3.8	55.1 ± 4.0	0.57
Sex (female, %)	85.7	90.9	80.0	0.48
Asthma onset (years)	37.3 ± 15.4	36.1 ± 4.7	38.6 ± 5.0	0.72
BMI (kg/m^2^)	25.0 (21.6–28.3)	21.7 (21.1–23.4)	28.3 (26.3–30.7)	0.0001
Smoking (ex, %)	23.8	27.3	20.0	0.70
Rhinitis (%)	90.5	90.9	90.0	0.94
Sinusitis (%)	57.1	63.6	50.0	0.53
FEV_1_/FVC (%)	68.6 ± 15.9	62.1 ± 4.4	75.8 ± 4.6	0.047
FVC (% predicted)	94.0 ± 20.1	96.9 ± 24.1	90.8 ± 15.2	0.50
FEV_1_ (% predicted)	76.6 ± 24.8	71.5 ± 7.5	82.1 ± 7.8	0.34
FeNO (ppb)	26.0 (15.0–36.5)	25.5 (18.8–43.3)	26.0 (11.5–36.5)	0.85
Blood eosinophils (/μL)	112.0 (6.5–219.0)	91.0 (0.0–191.0)	122.0 (30.3–459.3)	0.29
Serum total IgE (IU/mL)	50.0 (20.0–147.0)	50.0 (20.0–352.0)	51.0 (20.0–100.3)	0.52
Positive for serum specific IgE (%)	61.9	63.6	60.0	0.86
AQLQ (points)	4.3 ± 1.2	4.9 ± 0.3	3.7 ± 0.4	0.026
Exacerbations (/1 year)	1.0 (0.5–3.5)	1.0 (1.0–4.0)	1.0 (0.0–3.5)	0.28
High dose ICS-LABA (%)	100.0	100.0	100.0	1
LAMA (%)	57.1	54.5	60.0	0.80
LTRA (%)	100.0	100.0	100.0	1
mOCS (%)	47.6	63.6	30.0	0.12
Prednisolone (mg/day)	3.8 (2.5–5.0)	3.8 (2.5–5.0)	3.4 (0.8–5.6)	0.57
Biologics (%)	28.6	45.5	10.0	0.073
Omalizumab	14.3	18.2	10.0	
Mepolizumab	4.8	9.1	0.0	
Benralizumab	9.5	18.2	0.0	

Data are presented as mean ± SD or median (interquartile range, IQR). BMI, body mass index; FEV_1_, forced expiratory volume in 1 s; FVC, forced vital capacity; FeNO, fractional exhaled nitric oxide; AQLQ, Asthma Quality of Life Questionnaire; ICS, inhaled corticosteroid; LABA, long-acting β2 agonist; LAMA, long-acting muscarinic antagonist; LTRA, leukotriene receptor antagonist; mOCS, maintenance oral corticosteroid. ^†^ Comparisons between patients with BMI <25 kg/m^2^ and BMI ≥25 kg/m^2^ by Student's *t*-test, Wilcoxon rank-sum test for continuous variables, or χ^2^ test for dichotomous variables

Next, we grouped the patients into 2 groups according to their BMI levels: normal weight (BMI <25 kg/m^2^, *n* = 11) and overweight or obesity (BMI ≥25 kg/m^2^, *n* = 10). BMI of 25 kg/m^2^ was used as cut-off value according to the Japan Society for the Study of Obesity (JASSO).^8^ At baseline, patients with overweight/obesity had significantly higher FEV_1_/FVC and lower AQLQ scores than those with normal weight (Table 1). Blood eosinophils was ≥150/μL in 5 patients (50.0%) in the overweight/obesity group and 4 patients (36.4%) in the normal weight group. At 12 months after the procedure, 23.8% (*n* = 5) exhibited a decrease in BMI of at least 3%^9^ from the baseline assessment (normal weight, *n* = 2; overweight/obesity, *n* = 3; *P* = 0.53). Changes in BMI (kg/m^2^) were not significant in either group [normal weight, pre: 21.7 (21.1–23.4), post: 21.5 (19.9–22.4); *P* = 0.52, overweight/obesity, pre: 28.3 (26.3–30.7), post: 28.3 (25.8–31.2); *P* = 0.23]. The AQLQ scores were significantly improved in patients with overweight/obesity (Fig. 1a). In MANOVA, the degree of slopes significantly differed between the lines of overweight/obesity and normal weight in terms of the AQLQ scores (*P* = 0.01); these scores improved more in patients with overweight/obesity than those with normal weight. Meanwhile, the exacerbation frequency (Fig. 1b), FEV_1_ values (% predicted) (Fig. 1c), and FVC values (% predicted) (Fig. 1d) were significantly improved in those with normal weight. MANOVA revealed no significant differences in degree of slopes between normal weight and overweight/obesity in exacerbation frequency (*P* = 0.56), FEV_1_ values (% predicted) (*P* = 0.83), or FVC values (% predicted) (*P* = 0.48). None of the groups exhibited a significant decrease in the mOCS doses (Fig. 1e), probably due to the small sample size resulting from stratification by BMI. Even when we excluded 3 patients (overweight/obesity, *n* = 1; normal weight, *n* = 2) with a smoking history of 10 pack-years or more and FEV_1_/FVC ratio below 0.70, our main findings remained similar; the AQLQ scores were significantly improved in the overweight/obesity group alone (pre 3.86 ± 1.39, post 5.61 ± 1.53, *P* = 0.011). The normal weight group included patients who switched biologics during the study period (*n* = 3). This switch might have affected the outcomes; hence, these patients were excluded from the analysis. After the exclusion, the exacerbation frequency (*n* = 8, *P* = 0.063), FEV_1_ (% predicted) (*n* = 7, *P* = 0.17), and FVC (% predicted) (*n* = 7, *P* = 0.24) were no longer significantly improved in the normal weight group. Furthermore, Supplementary Fig. 3-5 depict the effects of baseline BMI on delta AQLQ, delta blood eosinophil counts, and delta FEV_1_ values (%predicted). Baseline BMI values were significantly associated with delta AQLQ even after adjustment with baseline (1) AQLQ (*P* = 0.038), (2) the presence of OCS (*P* = 0.023), (3) FEV_1_/FVC ratio below 0.70 (*P* = 0.005), or (4) at least four episodes of exacerbations in the past year (*P* = 0.023). When the analysis was confined to patients not taking any biologics in this study, the association remained significant between baseline BMI and the change in AQLQ scores (*r* = 0.546, *P* = 0.035). Baseline characteristics of patients with early-onset asthma and adult-onset asthma exhibiting overweight/obesity are shown in Supplementary Table 1.

**Fig. 1:**
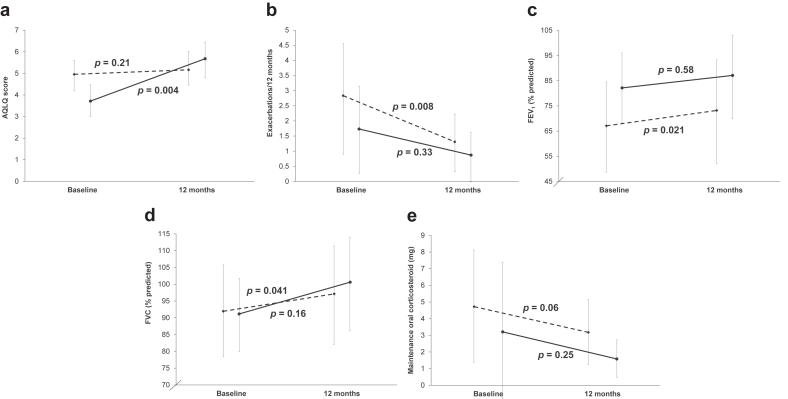
(a) Asthma Quality of Life Questionnaire (AQLQ) scores, (b) exacerbation frequency, (c) forced expiratory volume in 1 second (FEV_1_) (% predicted) values, (d) forced vital capacity (FVC) (% predicted) values, (e) maintenance oral corticosteroid doses (mOCS) at baseline and at 12 months after BT. Solid line represents patients with overweight/obesity, while the dotted line represents patients with normal weight. (a) (b) overweight/obese, *n* = 10; normal weight, *n* = 11. (c) (d) overweight/obese, *n* = 10; normal weight, *n* = 10. (e) Overweight/obese, *n* = 4; normal weight, *n* = 7. Means (95% confidence interval) are presented

This study identified a phenotype as the potential target for BT treatment to improve asthma QOL scores. This phenotype is characterized by female dominance, late-onset, overweight/obesity, low AQLQ, low-eosinophilic state under optimal treatment, and preserved pulmonary function. Its characteristics were overlapped with previously identified phenotypes of females with asthma and late-onset obesity^10^ with symptom predominance.^11^ In addition, the high BMI subgroup with higher FEV_1_/FVC had more asthma symptoms.^12^ The baseline AQLQ scores predicted the improvement in AQLQ scores in BT-treated patients (Supplementary Fig. 2), consistent with the previous reports.^1^, ^2^, ^3^

The impact of BMI on clinical effects after BT is discussed. While BMI values did not change significantly pre- and post- BT in either group, AQLQ scores significantly increased for patients with overweight/obesity than those with normal weight. Furthermore, in this study, baseline BMI values positively correlated with delta AQLQ, which remained significant after adjustment with baseline AQLQ, OCS, FEV_1_/FVC, or exacerbations. These results suggested the importance of BMI in the effectiveness of BT on patients with severe asthma. Meanwhile, baseline BMI values correlated with neither delta blood eosinophil counts nor delta FEV_1_ values (%predicted). These findings imply that improvements in AQLQ scores are not due to weight loss, suppression of eosinophilic inflammation, or improved lung function. The precise mechanism of BT effects in patients with severe asthma exhibiting overweight/obesity remains unknown, however, reduction of neuroendocrine epithelial cells, submucosal nerves, and airway smooth muscle-associated nerves may be involved.^13^ Hyperinsulinemia is more prevalent in obese individuals and can increase acetylcholine release and airway contraction by inhibiting M2 muscarinic receptors on airway parasympathetic nerves.^14^ Additionally, in mice models, leptin resistance in obesity might result in enhanced parasympathetic signaling and bronchoconstriction.^15^ Thus, in addition to airway smooth muscle reduction, nerve ablation *via* BT may contribute to favorable responses in patients with severe asthma exhibiting overweight/obesity. Otherwise, other potential mechanisms may be involved, including increased glucocorticoid receptor expression and heat shock protein expression.^16^

Currently, females with asthma exhibiting obesity are often steroid-resistant, and weight loss is considered to be the only optimal treatment for such patients.^6^ Our results newly demonstrated that BT could be a promising treatment option for the phenotype of overweight/obesity-related severe uncontrolled asthma. Because this phenotype is thought to have high symptom expression with discordantly low eosinophilic inflammation with appropriate treatment, it is possible that BT did not significantly improve objective indices. In contrast, patients with normal weight and low pulmonary function had non-significant improvement in exacerbation frequency and no improvement in the FEV_1_ values after excluding patients with biologics switch. The reason for the non-significant improvement of exacerbation frequency is probably because of the small sample size. Further studies are warranted to confirm the relevance of our findings. Further insight is required by accumulating evidence from large populations to select patients for BT treatment. In conclusion, BT treatment may obtain favorable QOL scores in patients with asthma exhibiting overweight/obesity with low QOL scores.

## Abbreviations

AQLQ, the Asthma Quality of Life Questionnaire; BMI, body mass index; BT, bronchial thermoplasty; FEV_1_, forced expiratory volume in 1 second; FVC, forced vital capacity; MANOVA, Multivariate Analysis of Variance; mOCS, maintenance oral corticosteroid; QOL, quality of life.

## Acknowledgements

The authors would like to thank Professor E. Juniper for allowing us to use the AQLQ Japanese translation for Japan. There is no funding for this study.

## Funding

There is no funding for this study.

## Availability of data and materials

The data analyzed during the current study are not publicly available because we did not obtain prior consent from the participants, but are available from the corresponding author on reasonable request.

## Author's contributions

KN collected, analyzed and interpreted the data and wrote the draft. CY and KM conceived and designed the study, collected, analyzed and interpreted the data. RI and ET collected and analyzed the data. TN wrote and edited the manuscript. HM revised the work critically. YN provided overall supervision and critically revised the manuscript.

## Ethics approval and consent to participate

This study was approved by The Ethics Committee of Osaka Red Cross Hospital (Registry ID: J-0183, UMIN000044207). Informed consent was obtained in the form of opt-out on the web-site.

## Consent for publication

All authors have approved the submission of this manuscript.

## Declaration of competing interest

The authors declare that they have no competing interests.
