# Supplementary material for: Effectiveness of bronchial thermoplasty in patients with asthma exhibiting overweight/obesity and low quality of life
Source: World Allergy Organ J. 2023 Mar 20;16(3):100756. doi: 10.1016/j.waojou.2023.100756 (PMC10040894; doi:10.1016/j.waojou.2023.100756)
Supplement: Multimedia component 1 [file mmc1.docx]

Supplementary Table 1. Baseline characteristics of patients with early-onset and adult-onset asthma exhibiting overweight/obesity.

|  | Overweight/obesity | |  |
| --- | --- | --- | --- |
|  | Early onset (*n* = 2) | Adult onset (*n* = 8) | *P* value^†^ |
| Age, years | 59.0 ± 7.1 | 54.1 ± 10.9 | 0.57 |
| Sex (female), % | 50.0 | 87.5 | 0.38 |
| Asthma onset, years | 15.0 ± 1.4 | 44.5 ± 10.8 | 0.006 |
| BMI, kg/m^2^ | 28.0 (25.6-30.4) | 28.3 (26.7-31.3) | 0.60 |
| Smoking (ex), % | 0.0 | 25.0 | 1.0 |
| Rhinitis, % | 100.0 | 87.5 | 1.0 |
| Sinusitis, % | 100.0 | 37.5 | 0.44 |
| Hypertension, % | 0.0 | 12.5 | 1.0 |
| Diabetes mellitus, % | 0.0 | 12.5 | 1.0 |
| FEV_1_/FVC, % | 62.3 ± 9.5 | 79.2 ± 8.9 | 0.044 |
| FVC, % predicted | 77.2 ± 1.8 | 94.2 ± 15.1 | 0.17 |
| FEV_1_, % predicted | 57.1 ± 10.0 | 88.4 ± 15.7 | 0.031 |
| FeNO, ppb | 44.0 (30.0–58.0) | 17.0 (10.0–29.0) | 0.079 |
| Blood eosinophils, /µL | 468.5 (450.0–487.0) | 81.0 (18.8–156.8) | 0.12 |
| Serum total IgE, IU/mL | 52.5 (20.0–85.0) | 51.0 (24.0–104.8) | 0.60 |
| Positive for serum specific IgE, % | 50.0 | 62.5 | 1.0 |
| AQLQ, points | 3.2 ± 2.9 | 3.9 ± 1.0 | 0.53 |
| Exacerbations, per year | 0.5 (0.0–1.0) | 1.5 (0.0–4.5) | 0.42 |
| High dose ICS-LABA, % | 100.0 | 100.0 | − |
| LAMA, % | 50.0 | 62.5 | 1.0 |
| LTRA, % | 100.0 | 100.0 | − |
| mOCS, % | 50.0 | 25.0 | 1.0 |
| Prednisolone, mg/day | 3.8 (3.8–3.8) | 3.1 (0.0–6.3) | 0.65 |
| Biologics (Omalizumab), % | 0.0 | 12.5 | 1.0 |

Data are presented as mean ± SD or median (interquartile range, IQR). BMI, body mass index; FEV_1_, forced expiratory volume in one second; FVC, forced vital capacity; FeNO, fractional exhaled nitric oxide; AQLQ, Asthma Quality of Life Questionnaire; ICS, inhaled corticosteroid; LABA, long-acting β2 agonist; LAMA, long-acting muscarinic antagonist; LTRA, leukotriene receptor antagonist; mOCS, maintenance oral corticosteroid. ^†^ Comparisons between patients with early and adult onset by Student’s *t*-test, Wilcoxon rank-sum test for continuous variables, or χ^2^ test for dichotomous variables.
