# Supplementary material for: Effectiveness of bronchial thermoplasty in patients with asthma exhibiting overweight/obesity and low quality of life
Source: World Allergy Organ J. 2023 Mar 20;16(3):100756. doi: 10.1016/j.waojou.2023.100756 (PMC10040894; doi:10.1016/j.waojou.2023.100756)
Supplement: Multimedia component 2 [file mmc2.pdf]

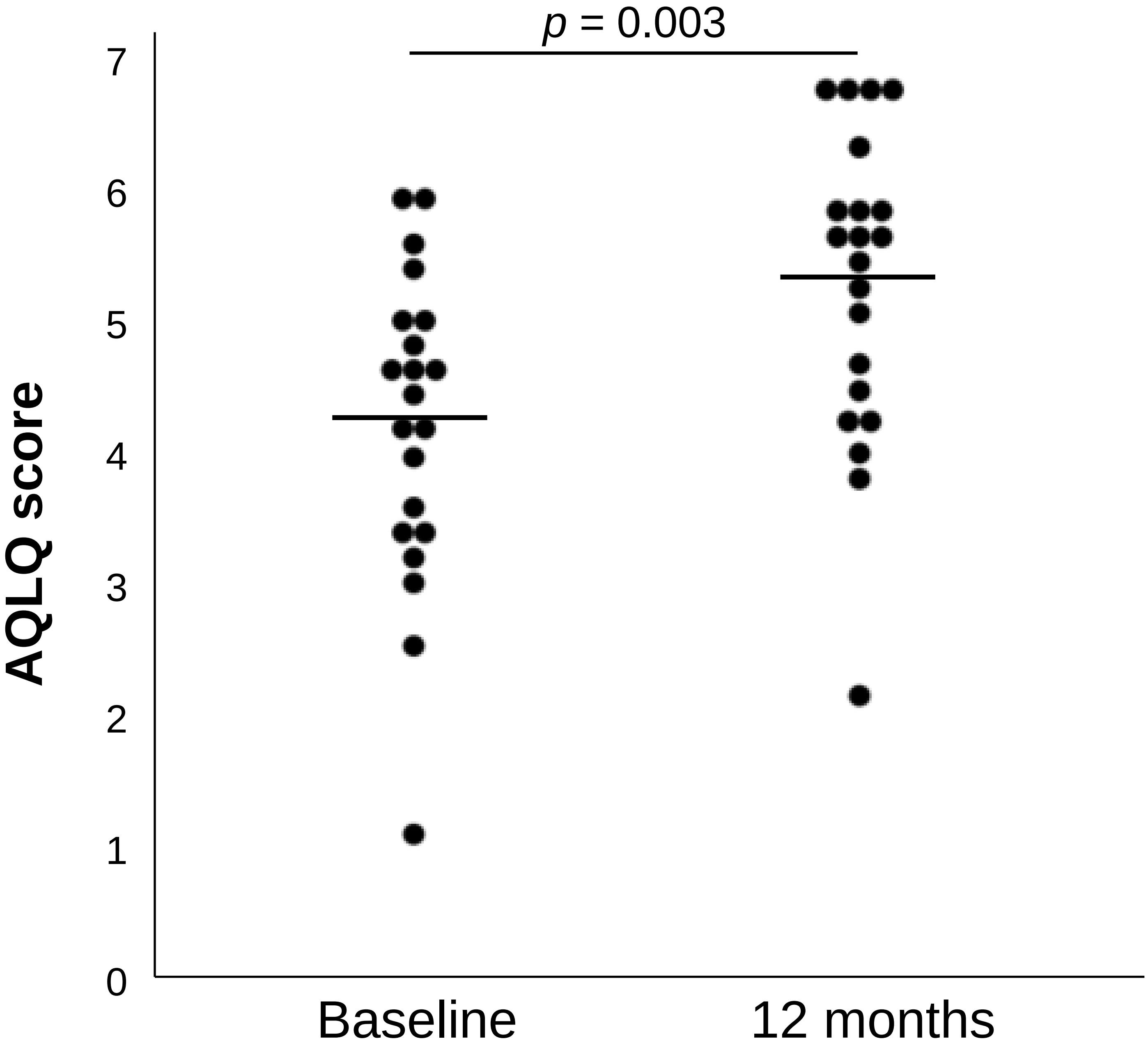

Supplementary Fig. 1a

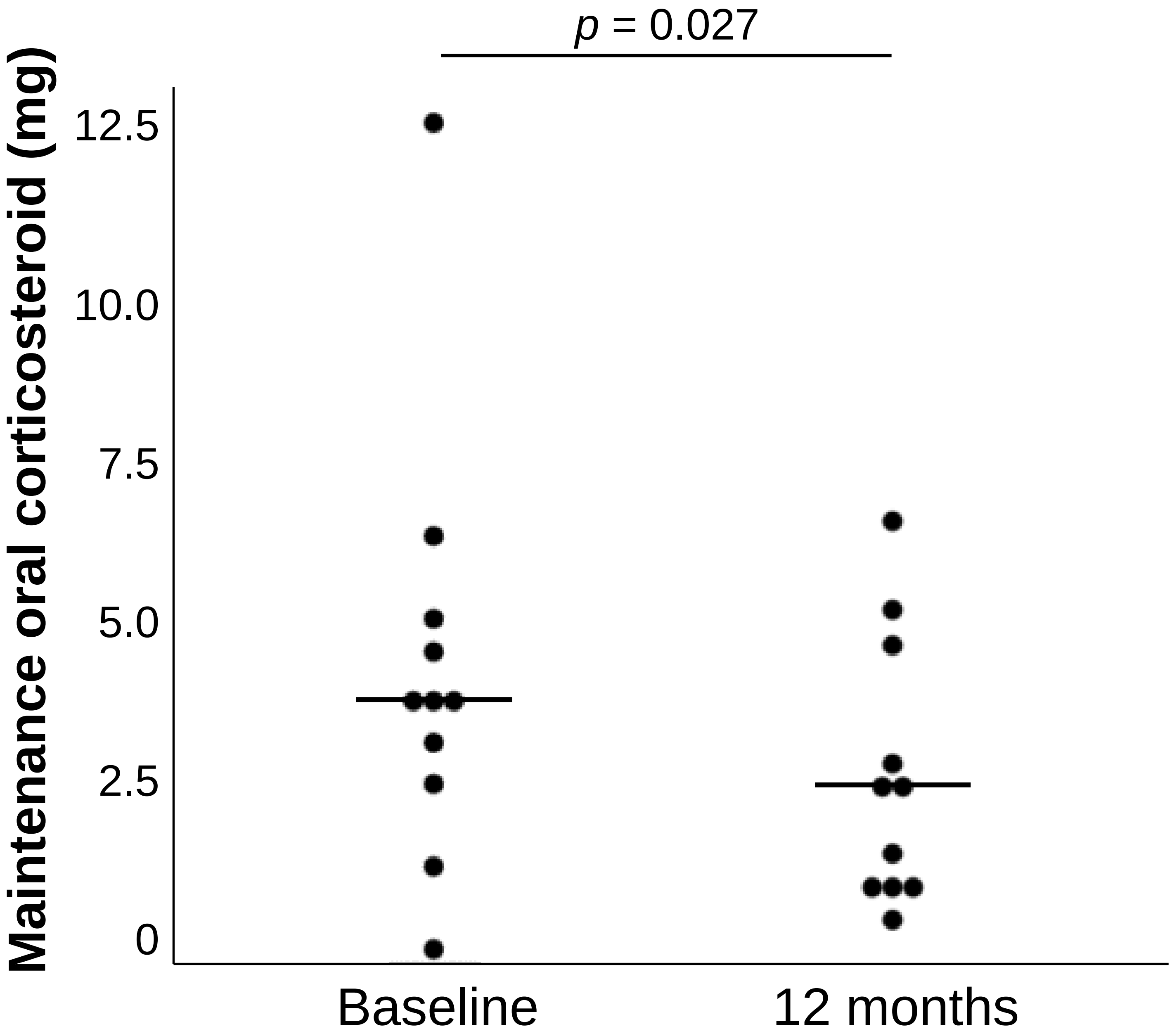

Supplementary Fig. 1b

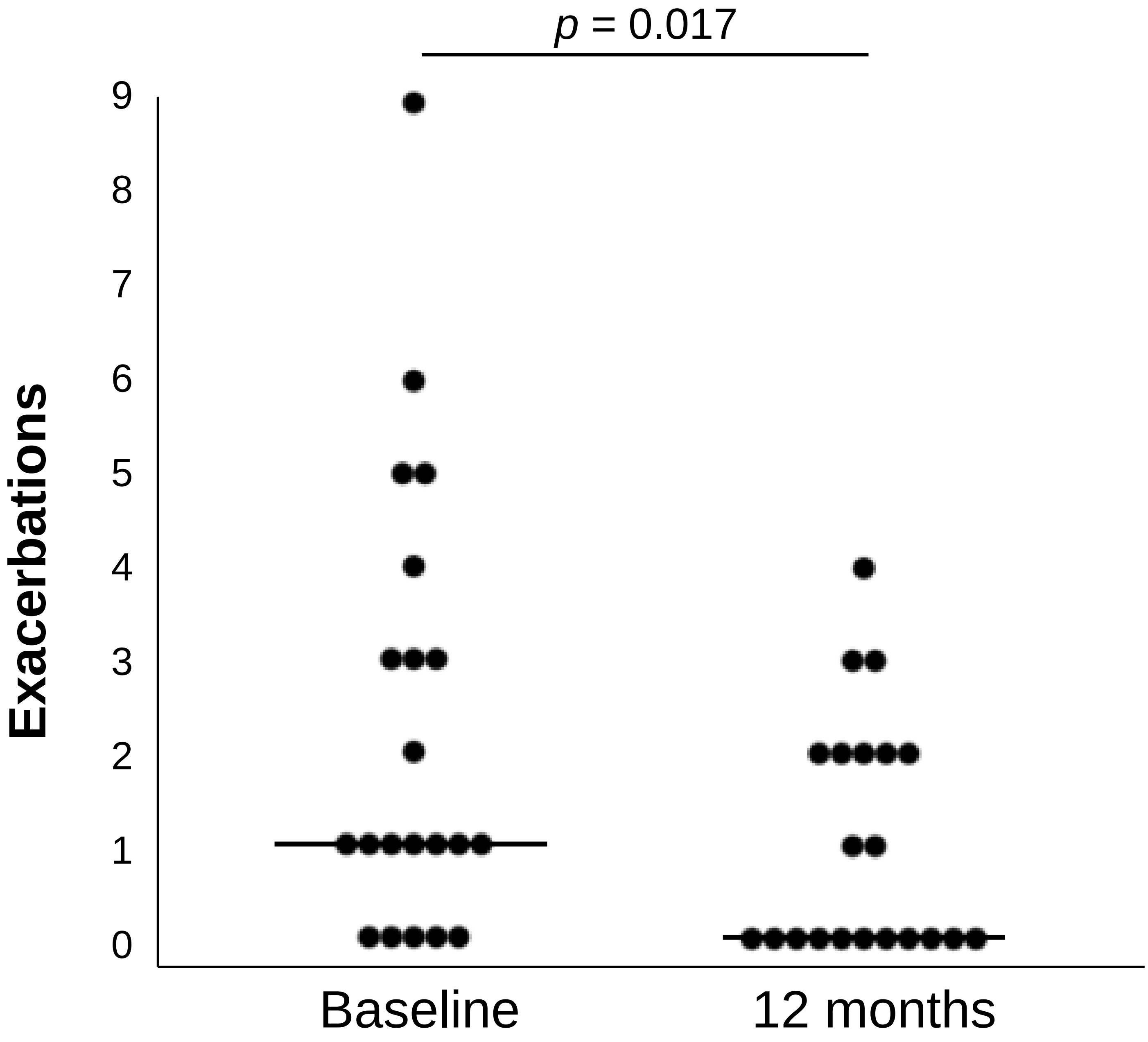

Supplementary Fig. 1c

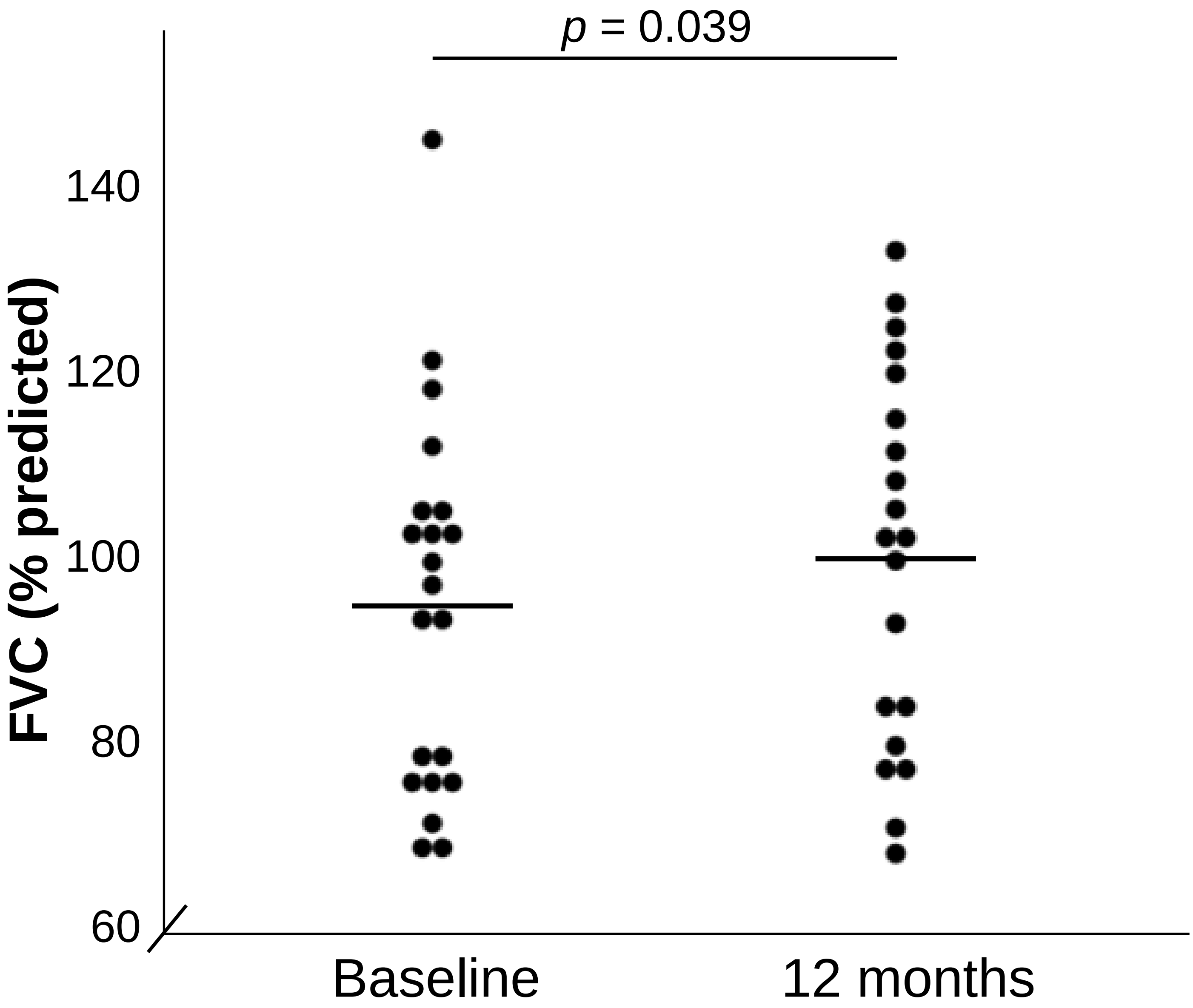

Supplementary Fig. 1d

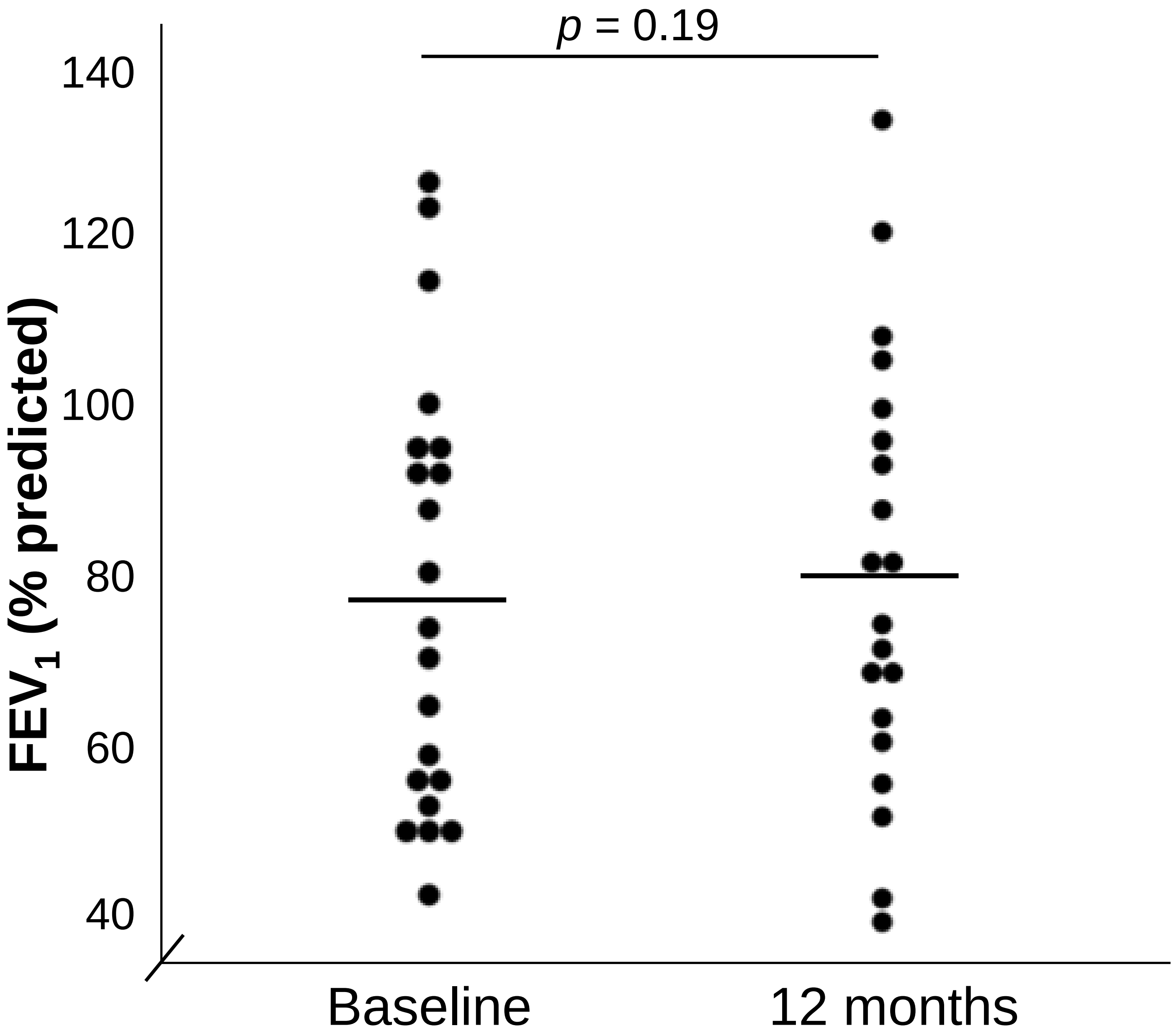

Supplementary Fig. 1e

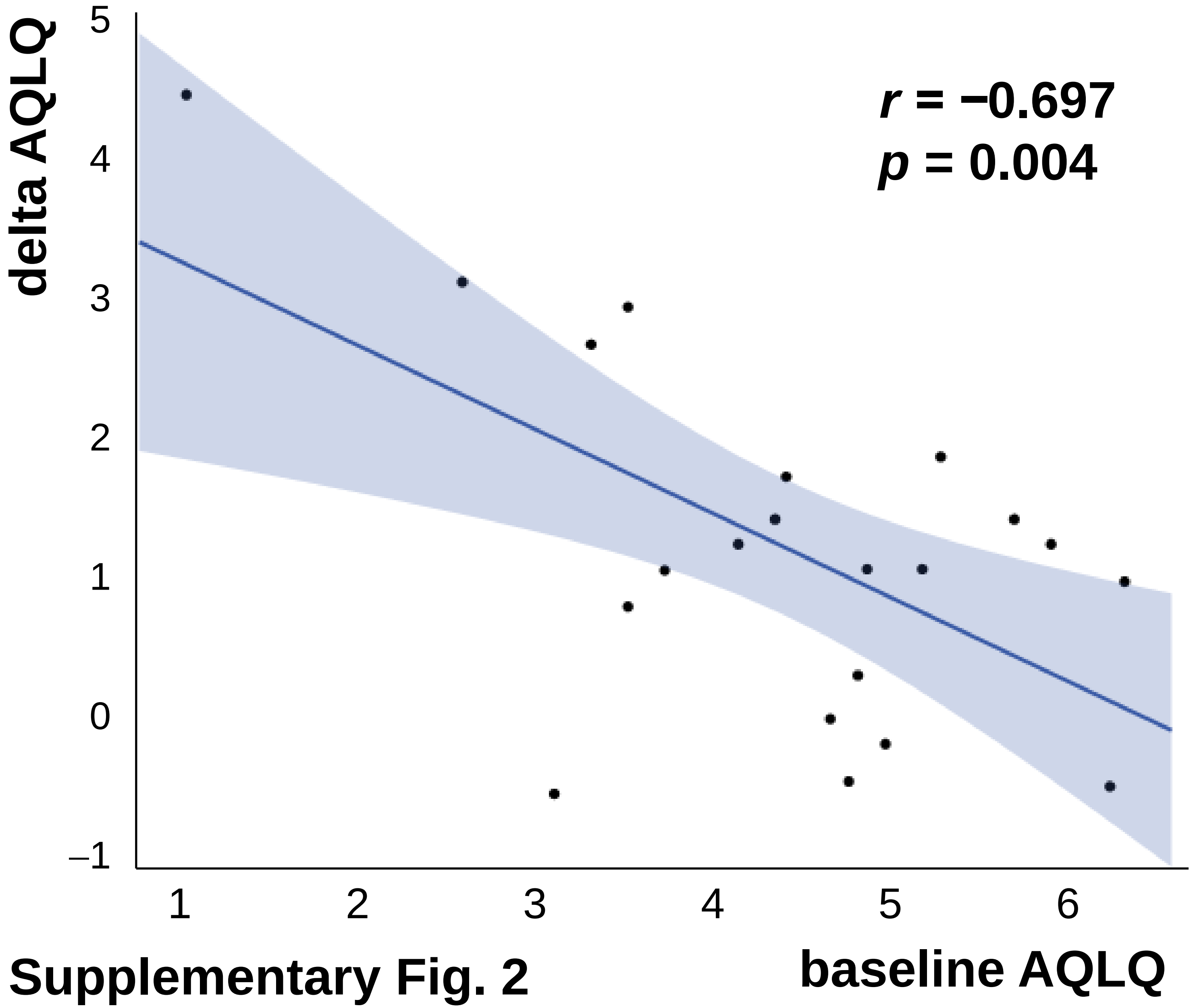

Supplementary Fig. 2

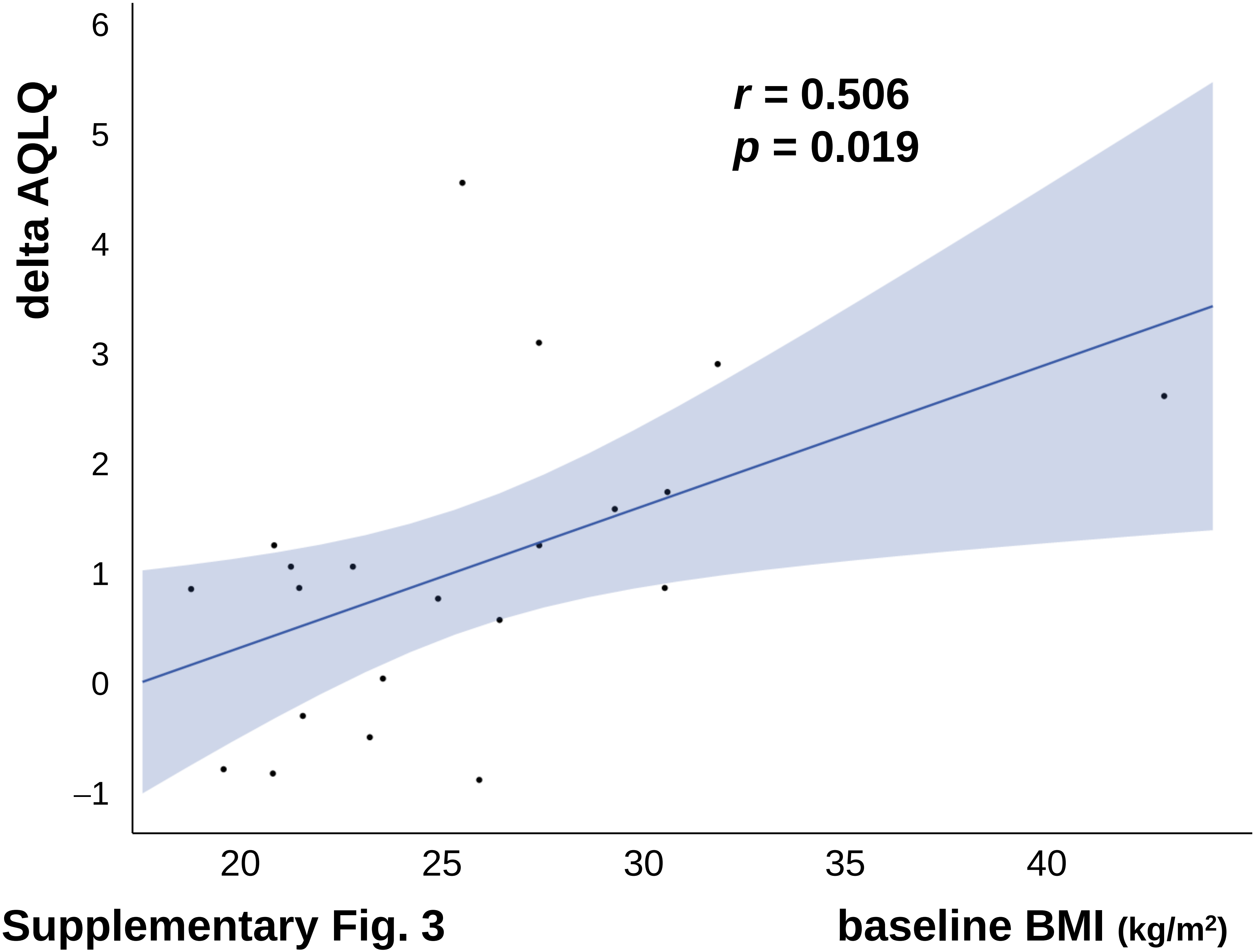

Supplementary Fig. 3

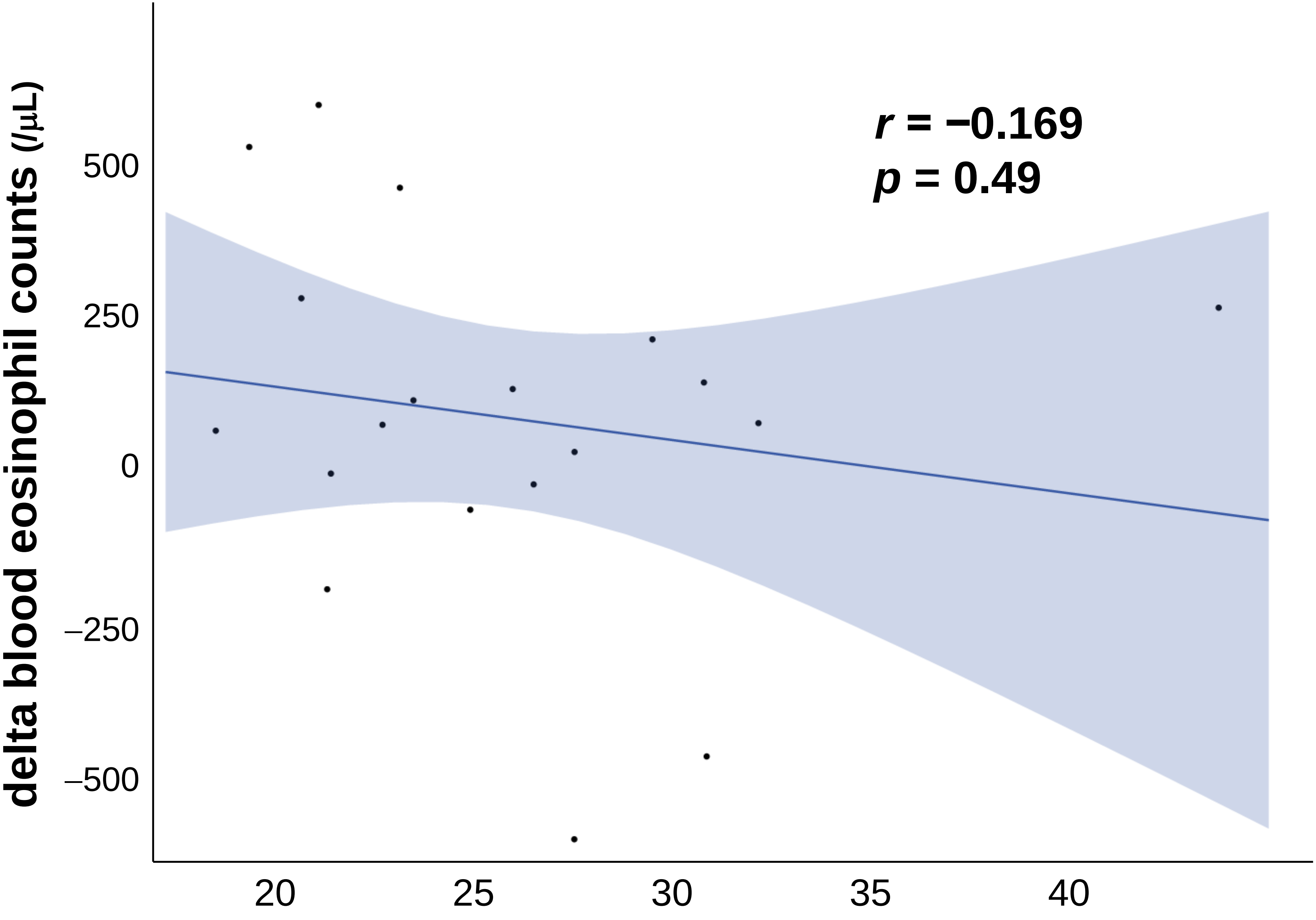

Supplementary Fig. 4

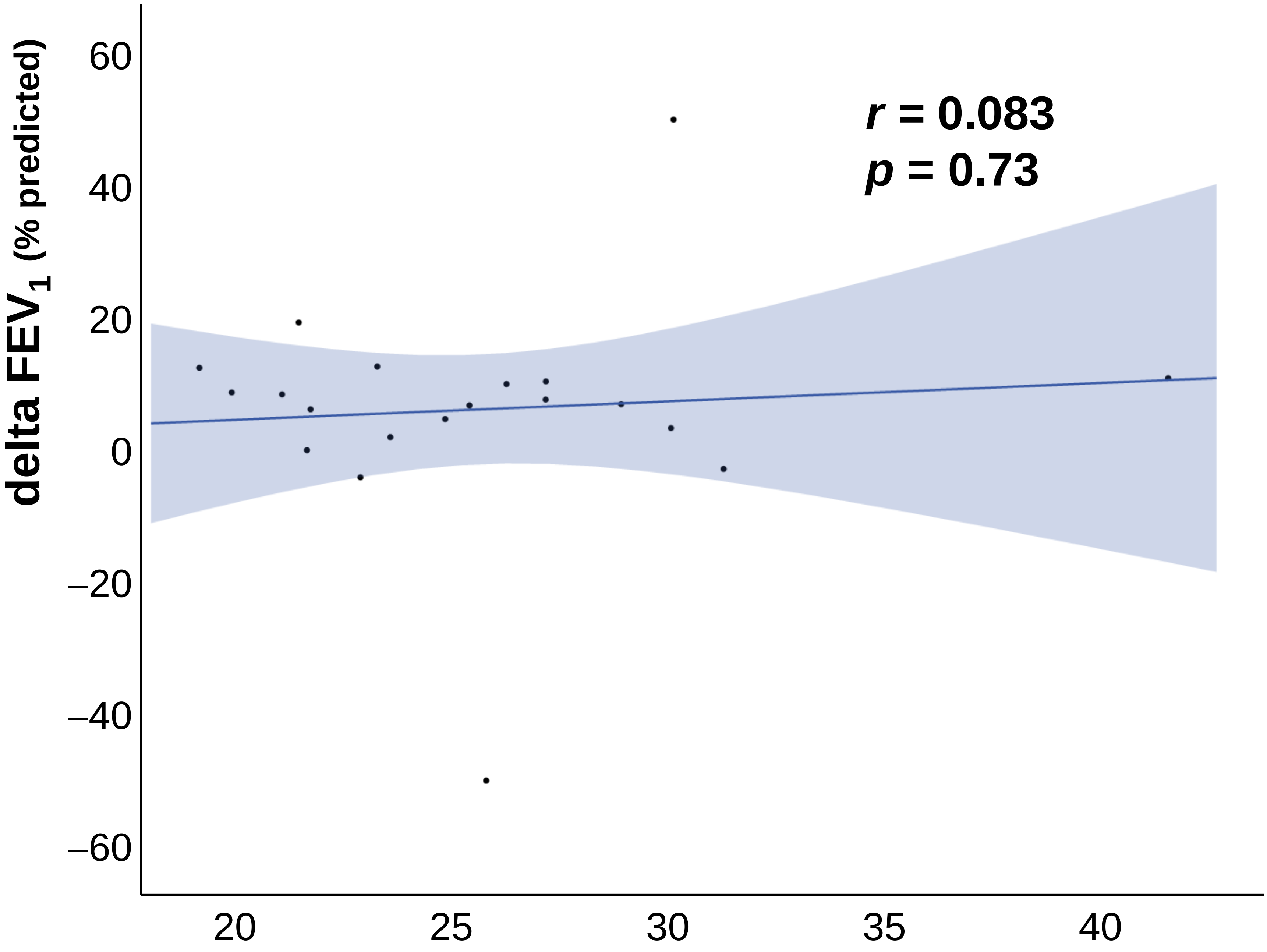

Supplementary Fig. 5
